# Markedly T2-Hypointense Clival Plasmacytoma With Light Chain Deposition Disease: A Case Report

**DOI:** 10.7759/cureus.76289

**Published:** 2024-12-23

**Authors:** Tiffany Shamas, Conner D Reynolds, Ryan R Garcia, Rosalie Doescher, Deborah Fuchs, Samuel N Rogers

**Affiliations:** 1 Department of Medical Imaging, University of Arizona College of Medicine - Tucson, Tucson, USA; 2 Department of Pathology, University of Arizona College of Medicine - Tucson, Tucson, USA

**Keywords:** clivus, light chain deposition disease, mri - magnetic resonance imaging, plasmacytoma, t2 hypointense

## Abstract

Plasmacytomas are rare monoclonal neoplastic plasma cell proliferations in soft tissue or bone, with clival plasmacytomas being extremely rare and occasionally presenting with light chain deposition disease (LCDD). While imaging findings for clival plasmacytomas have shown variable T2 signal characteristics, complete T2 signal loss has not been previously reported.

We present a case of a 61-year-old female found to have a 1.9 cm expansile lytic lesion in the clivus on CT. MRI revealed intermediate T1 signal and near-complete T2 signal loss. Histopathology demonstrated kappa-restricted plasma cells with extracellular light chain deposition, while bone marrow biopsy showed 5-10% plasma cells without evidence of multiple myeloma. The patient was diagnosed with a solitary plasmacytoma of the clivus with associated LCDD and underwent endoscopic resection and radiotherapy, showing a favorable initial response at a seven-month follow-up.

This case highlights the importance of considering plasmacytoma with associated LCDD for marked T2 hypointense, enhancing skull base lesions. The combination of plasmacytoma and LCDD may explain the unusual imaging characteristics, underscoring the need for comprehensive diagnostic approaches in patients presenting with unusually hypointense clival lesions.

## Introduction

Light chain deposition disease (LCDD) is a rare disorder characterized by the deposition of monoclonal immunoglobulin light chains in various organs, most commonly the kidneys [1,2]. It belongs to the broader category of monoclonal immunoglobulin deposition diseases (MIDD) and is distinct from amyloidosis due to the non-fibrillar nature of the deposits [3]. LCDD often occurs in association with plasma cell dyscrasias, including multiple myeloma and monoclonal gammopathy of undetermined significance (MGUS) [2]. Plasmacytomas are discrete tumors of neoplastic monoclonal plasma cells that can arise in bone (solitary plasmacytoma of bone (SPB)) or soft tissue (extramedullary plasmacytoma) [4]. SPB comprises approximately 2-5% of plasma cell tumors and is considered an intermediary phase between MGUS and multiple myeloma [5]. The pathogenesis of LCDD involves the overproduction and tissue deposition of structurally abnormal immunoglobulin light chains, predominantly kappa light chains [2]. These deposits can affect multiple organs, leading to various clinical manifestations. While the kidneys are almost universally involved, extrarenal deposition can occur in the heart, liver, and nervous system [6].

Clival plasmacytomas are extremely rare, with less than 10 reported cases in the literature [7]. They represent a very small subset of intracranial plasmacytomas, which themselves account for about 1% of all cerebral tumors [8]. These lesions were often present with kappa or lambda light chain restriction, which can be detected through in situ hybridization (ISH), establishes monoclonality, and helps differentiate plasmacytoma from a reactive polymorphous plasma cell population. Establishing restriction of light chains in a plasmacytoma does not establish the diagnosis of LCDD, as the light chains are intracellular and only very rarely deposited extracellularly, as is required with LCDD.

Imaging characteristics of clival plasmacytomas typically include iso-dense lytic lesions on computed tomography (CT) and iso-to-hypointense signals on T1-weighted (T1W) magnetic resonance imaging (MRI) sequences with enhancement. On T2-weighted (T2W) sequences, these lesions are variable and may sometimes be intermediate or low in signal, though we are not aware of any cases that have shown near-complete signal loss on T2W images [9].

The association of plasmacytoma with LCDD presents unique diagnostic and therapeutic challenges. Understanding the interplay between these conditions is crucial for accurate diagnosis, appropriate management, and monitoring of disease progression. This case report aims to highlight the rare occurrence of a clival plasmacytoma with suspected associated LCDD, emphasizing the importance of considering this entity in the differential diagnosis of clival lesions with atypical imaging characteristics.

## Case presentation

We present a case of a 61-year-old female with a known vestibular schwannoma in the right internal auditory canal. At the time of presentation, the patient was asymptomatic and did not present with diplopia, facial pain/pressure, headache, seizures, or any focal neurological deficits. On examination, the patient’s vitals were stable with intact cognitive function.

**Figure 1:**
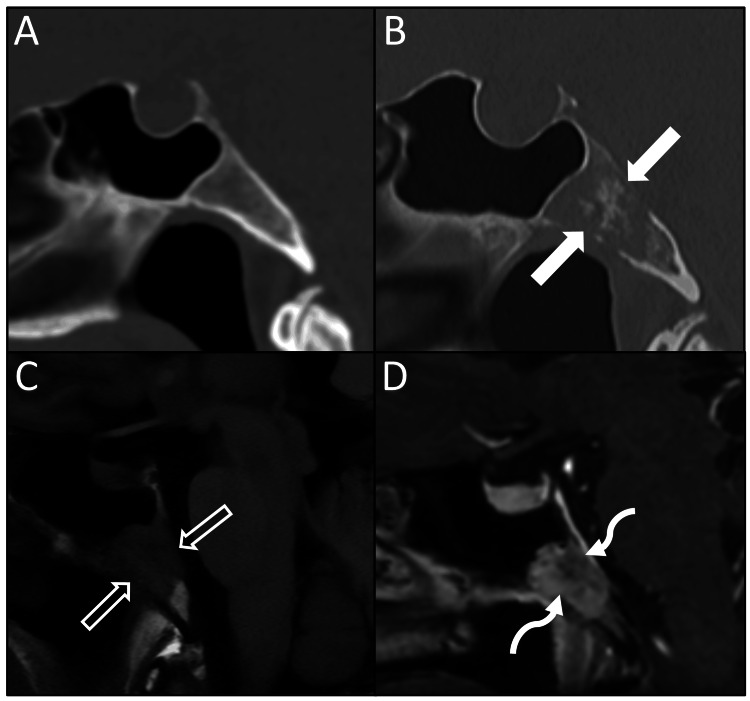
Progressive development of clival lesion in a patient with known vestibular schwannoma Surveillance CT for known vestibular schwannoma demonstrates normal clivus on sagittal reconstruction (A). Four years later, surveillance CT demonstrates an expansile lytic lesion (B, solid arrows) at the clivus on sagittal reconstruction. Follow-up sagittal MRI images demonstrate the mass to be marrow-replacing with T1 hypointensity (C, open arrows) and heterogeneous enhancement (D, curved arrows).

**Figure 2:**
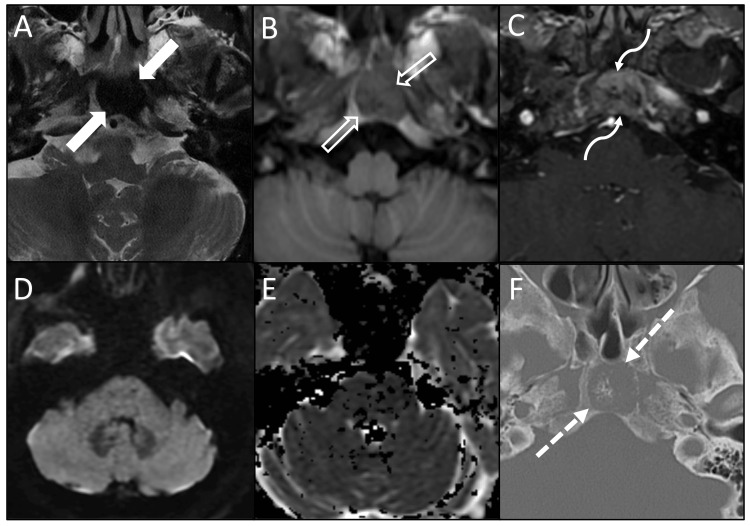
Multimodality imaging features of a clival mass: MRI and CT characteristics Additional axial MRI images of the clival mass demonstrate complete loss of signal on T2 (A, solid arrows), T1 marrow replacement (B, open arrows), heterogeneous enhancement (C, curved arrows), and no signal on DWI or ADC (D, E). Comparison axial CT demonstrates the clival lesion to be lytic with residual internal bony trabecula (F, dashed arrows).

The patient was incidentally found to have a 1.9 cm lytic lesion within the clivus on CT with a small amount of internal calcifications in the central portion of the lesion (Figure 1). MRI of the brain was then obtained, which showed a well-circumscribed lesion in the midline clivus measuring up to 2.0 cm. The lesion demonstrated intermediate signal intensity on T1W images with heterogeneous enhancement and complete signal loss on T2W images (Figure 2). There was no definite evidence of intracranial extension. The lesion did not demonstrate diffusion restriction or high signal on fluid-attenuated inversion recovery (FLAIR) sequences.

The patient underwent an endoscopic endonasal transsphenoidal approach for resection of the tumor. The clival mass consisted of 1.1 x 0.5 x 0.2 cm red-tan fibrous soft tissue with erosion and replacement of the clival bone.

**Figure 3:**
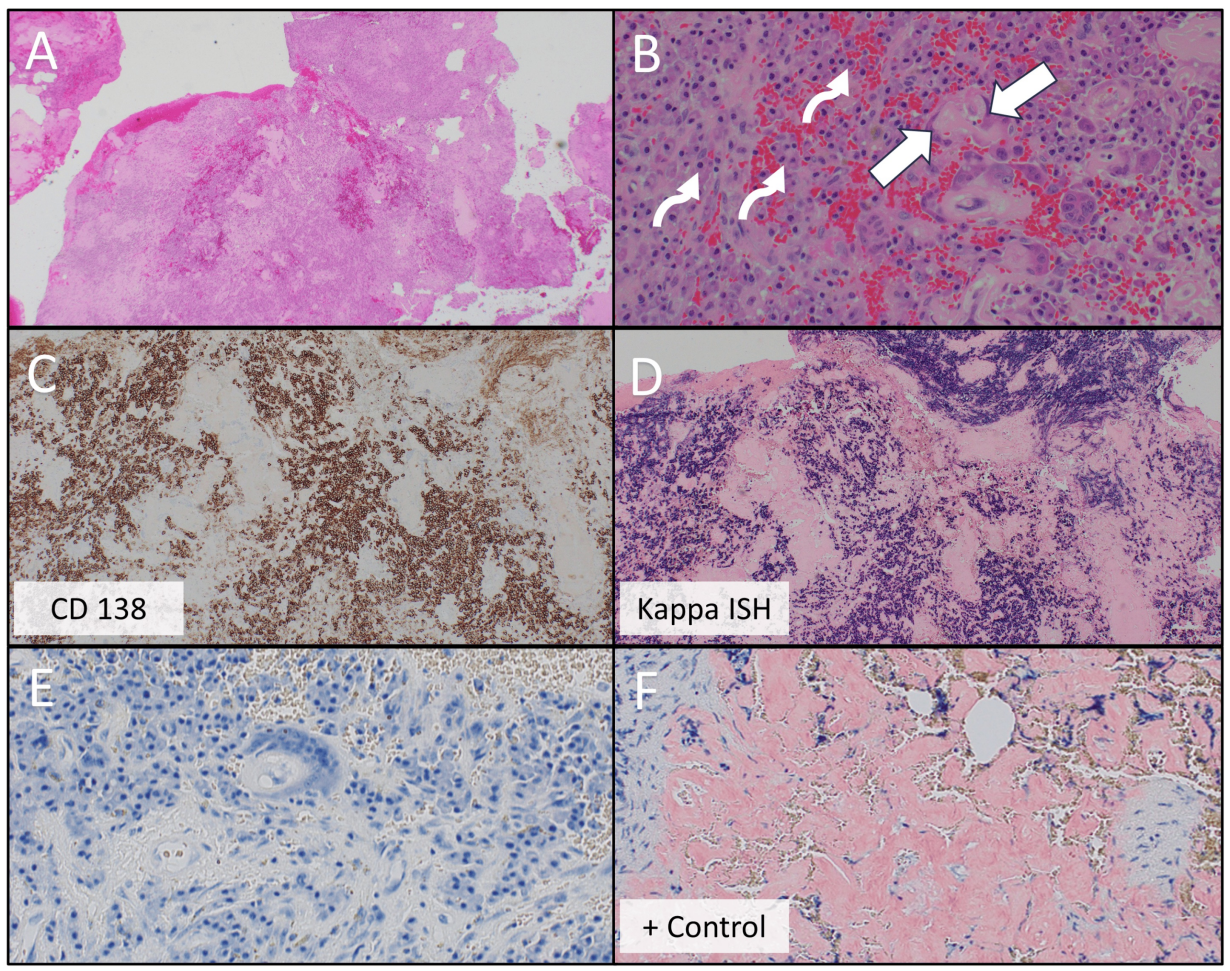
Histopathological and immunohistochemical features of plasma cell neoplasm with extracellular deposits Cellular infiltrate (A), high-power magnification showing extracellular amorphous material (B, solid arrows) along with sheets of mature plasma cells that are CD138 positive (B, curved arrows, C). In situ hybridization with monoclonal plasma cells expressing kappa light chain (D). Congo red stain for amyloid on two blocks was negative compared to the control (E, F). Magnification: A) 20x; B) 200x; C) 40x; D) 40x; E) 200x; F) 200x

Histopathological examination revealed sheets of plasma cells that were CD138-positive and kappa-restricted by ISH, consistent with a plasma cell neoplasm. Notably, abundant extracellular amorphous material was observed, initially suspicious for amyloid, although the Congo red stain was negative (Figure 3). Congo red stain was repeated, which was again negative, and mass spectrometry was performed, confirming a predominance of peptides in the extracellular material associated with kappa immunoglobulin light chains, consistent with kappa LCDD. There was no evidence of an abnormal B-cell proliferation to suggest a lymphoplasmacytic lymphoma.

To rule out systemic involvement, a bone marrow biopsy was performed. The biopsy showed a normocellular marrow with trilineage hematopoiesis and 5-10% predominantly kappa light-chain-positive plasma cells. Immunohistochemistry with CD138 confirmed 5-10% plasma cells focally forming small clusters, but no sheets of plasma cells were present. ISH demonstrated a kappa-to-lambda ratio of approximately 9:1. Flow cytometry revealed a minor polytypic plasma cell population with a subset aberrantly expressing CD56. Importantly, the multiple myeloma ISH panel was normal.

Laboratory investigations (Table 1) revealed no evidence of hypercalcemia, renal insufficiency, anemia, or bone lesions, which would suggest conversion to multiple myeloma. Complete blood count showed normal values: WBC of 6.7 K/uL, RBC of 4.64 M/uL, hemoglobin of 13.7 g/dL, hematocrit of 41.5%, and platelets of 355 k/uL. The comprehensive metabolic panel was largely unremarkable with creatinine of 0.9 mg/dL, blood urea nitrogen (BUN) of 18.0 mg/dL, calcium of 9.7 mg/dL, total protein of 8.0 g/dL, and albumin of 4.2 g/dL. Liver function tests and electrolytes were within normal limits, except for an elevated glucose of 173 mg/dL. Beta-2 microglobulin was 0.49 g/dL. Serum protein electrophoresis showed no monoclonal proteins, and IgG, IgA, and IgM levels were normal. However, the free kappa light chain was elevated at 81.37 mg/L, with a normal free lambda light chain of 12.26 mg/L, resulting in an elevated kappa-to-lambda ratio of 6.64.

**Table 1:** Laboratory findings in a patient with suspected plasmacytoma and light chain deposition disease AST: aspartate aminotransferase; BUN: blood urea nitrogen

Test	Result	Reference range
WBC	6.7	4.5-11.0 K/uL
RBC	4.64	3.7-5.4 M/uL
Hemoglobin	13.7	11.5-16.0 g/dL
Hematocrit	41.5	35-48%
Platelets	355	140-450 k/uL
Na	137	136-145 mmol/L
K	4.5	3.5-5.1 mmol/L
Cl	101	98-107 mmol/L
CO_2_	25	21-32 mmol/L
Creatinine	0.9	0.55-1.02 mg/dL
BUN	18.0	7-18 mg/dL
Glucose	173 (high)	74-107 mg/dL
Calcium	9.7	8.5-10.1 mg/dL
Total protein	8.0	6.4-8.2 g/dL
Albumin	4.2	3.5-5.0 g/dL
Alk Phos	84	46-116 U/L
AST	24	15-37 U/L
IgG	913	694-1618 mg/dL
IgA	288	81-463 mg/dL
IgM	96	48-271 mg/dL
Beta-2 microglobulin	0.49	0.20-0.52 g/dL
Free kappa light chain	81.37 (high)	3.30-19.40 mg/L
Free lambda light chain	12.26	5.71-26.30 mg/L
Free kappa-to-lambda ratio	6.64 (high)	0.26-1.65

These findings, in the absence of other bone lesions or end-organ damage, were consistent with a solitary plasmacytoma with minimal bone marrow involvement (<10%), rather than multiple myeloma.

The patient was ultimately diagnosed with a solitary plasmacytoma of the clivus with associated LCDD and limited bone marrow involvement. She was treated with four weeks of radiotherapy, receiving a total dose of 4000 cGy. At seven months post-treatment, follow-up CT showed no evidence of recurrent or metastatic disease.

Due to the high-risk nature of the disease, the patient was recommended to continue follow-up visits and laboratory tests every six months for the next year to monitor for potential progression to multiple myeloma.

This case highlights the rare association of plasmacytoma with LCDD, which may explain the unusual imaging characteristics. It emphasizes the importance of comprehensive evaluation, including bone marrow biopsy and serum-free light chain analysis, to accurately diagnose and differentiate solitary plasmacytoma from multiple myeloma, particularly when presenting with atypical features such as marked T2 hypointensity on MRI.

## Discussion

This case uniquely demonstrates a rare presentation of clival plasmacytoma with suspected associated local LCDD, which has not been previously described in the literature. Clinically, patients with clival plasmacytoma tend to present with headaches, diplopia, or other focal neurologic deficits [10]. Additionally, the markedly decreased signal on T2W MRI sequences is an atypical finding for plasmacytomas and warrants further discussion.

LCDD is characterized by the accumulation of monoclonal immunoglobulin light chains in various tissues [2]. While LCDD most commonly affects the kidneys, extrarenal involvement, including in the central nervous system, has been reported [11]. In this case, the extracellular deposition of light chains remained isolated to the site of the plasmacytoma.

The deposition of light chains in tissues can lead to structural changes that may alter imaging characteristics, which may explain the highly unusual near-complete signal void on T2W sequences. We propose that the extensive deposition of light chains in the tumor microenvironment could explain this atypical imaging appearance. Marked T2 hypointensity in LCDD has been reported elsewhere in the central nervous system [12]. The accumulation of light chains may increase the protein concentration within the lesion, leading to T2 signal loss due to decreased free water content [13]. Additionally, the fibrillar nature of light chain deposits could contribute to a more organized tissue structure, further reducing T2 signal intensity.

Overall, solitary clival lesions are uncommon with a relatively broad range of differential diagnoses. MRI can be utilized to narrow down this list by evaluating their signal intensity on T1W and T2W sequences. Additional clival lesions with decreased T2 signal include amyloidoma, lymphoma, ossifying fibroma, sphenoid sinus fungus ball and mucocele, fibrous dysplasia, sinonasal neuroendocrine carcinoma and adenocarcinoma, and metastasis. The lymphoproliferative disease may be the hardest to differentiate from plasmacytoma, with similar imaging features. Amyloidoma, as well as fungus balls and mucoceles, generally show little to no enhancement on T1, contrary to plasmacytoma. Ossifying fibroma shows more coarse bony productive change on CT, and fibrous dysplasia usually displays the characteristic ground glass appearance both on CT and T2. Sinonasal tumors may usually be traced to their mucosal origins and are generally more heterogeneous than plasmacytoma [14,15].

The association of plasmacytoma with LCDD is particularly rare, with only a few cases reported in the literature [2]. This combination presents unique diagnostic challenges, as the imaging features may differ from those typically seen in isolated plasmacytomas. Our case highlights the importance of considering LCDD in the differential diagnosis of clival lesions demonstrating marked T2 hypointensity.

The presence of increased free serum light chains in plasmacytomas has been associated with an increased risk of progression to multiple myeloma [2]. It is unclear whether or not localized LCDD in association with plasmacytoma may have similar prognostic implications. In our patient's case, the combination of plasmacytoma with suspected LCDD necessitated a comprehensive approach to treatment, including surgical resection, radiotherapy, and close lifelong follow-up for potential progression to multiple myeloma.

In conclusion, this case expands our understanding of the radiological presentation of clival plasmacytomas and highlights the potential role of light chain deposition in altering imaging characteristics. Clinicians, radiologists, and pathologists should be aware of this possibility when encountering clival lesions with atypical imaging features, particularly marked T2 hypointensity. Further research is needed to elucidate the relationship between light chain deposition and MRI signal characteristics in plasma cell disorders.

## Conclusions

This case highlights the importance of including plasmacytoma in the differential diagnosis for clival tumors that demonstrate T2 hypointensity on MRI, with marked hypointensity a possible marker of LCDD that warrants further research.
